# Pseudoseptic Arthritis in a Patient With Psoriasis

**DOI:** 10.7759/cureus.19185

**Published:** 2021-11-01

**Authors:** Jorge Verdecia, Karishma P Ramsubeik, Malleswari Ravi

**Affiliations:** 1 Internal Medicine, University of Florida College of Medicine – Jacksonville, Jacksonville, USA; 2 Rheumatology, University of Florida College of Medicine – Jacksonville, Jacksonville, USA; 3 Infectious Disease, University of Florida College of Medicine – Jacksonville, Jacksonville, USA; 4 Infectious Disease, University of Florida, Jacksonville, Jacksonville, USA

**Keywords:** culture negative arthritis, acute bacterial arthritis, psoriasis, septic arthritis, pseudoseptic arthritis

## Abstract

A 42-year-old male with a history of untreated psoriasis and a previous episode of presumed left knee septic arthritis developed sudden onset of left knee pain, swelling, and a moderate effusion. The pathogen could not be isolated despite extensive inflammation seen in synovial fluid (SF) and synovial tissue biopsy. Whether this is culture-negative septic arthritis or pseudo-septic arthritis is the enigma, given the limited sensitivity of current available SF microbiologic testing. We present a challenging and stimulating case with no current guidelines for an optimal empiric antibiotic regimen or anti-inflammatory therapy.

## Introduction

Acute bacterial arthritis or septic arthritis [SA] is a condition where joints are infected with microorganisms, and potential destruction of the joint ensues. SA carries high mortality and morbidity, especially in those with significant comorbidities. The knee is the most commonly involved [1]. SA of native joints is usually from hematogenous seeding of the vascular synovial membrane from overt or occult bacteremia [1,2]. Pseudoseptic arthritis is an acute inflammatory monoarthritis with a sterile synovial gram stain and culture and can clinically mimic septic arthritis.

## Case presentation

**Figure 1:**
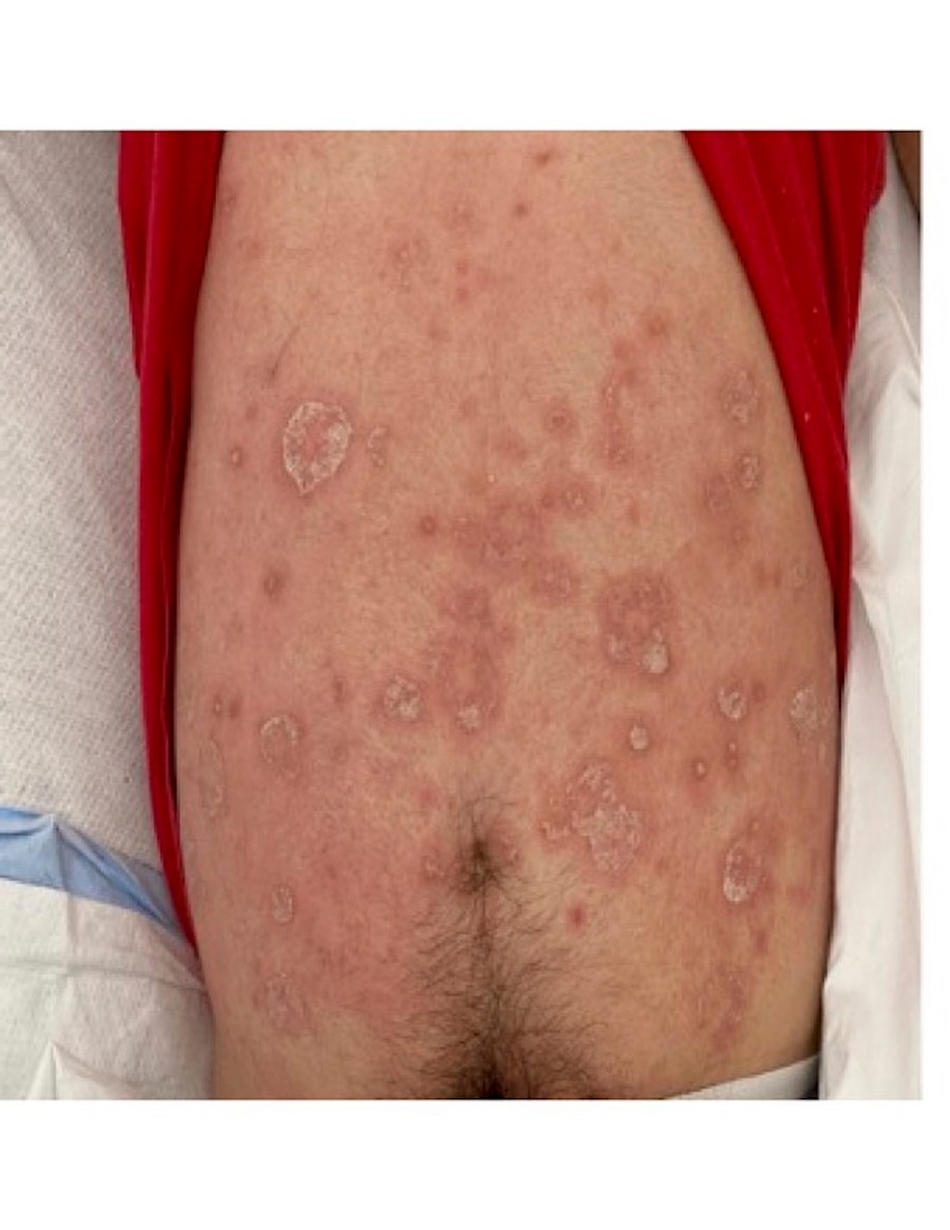
Skin lesions consistent with psoriasis

The patient is a 42-year-old male from Mexico who has been living in the United States for 20 years that presented with sudden onset of left knee pain and swelling for nine days. He has a past medical history of untreated psoriasis for several years and presumed septic arthritis of the left knee one year ago. He underwent debridement followed by a 4-week empiric intravenous vancomycin therapy at an outside facility. He described the pain as dull, aching, constant, moderate, and without radiation. The pain was made worse with the movement of the extremity. He denied fevers, trauma, and illicit drug use. He reported no sexual activity for one year, lived alone, and did not have any pets. He worked as a floor technician in a school. There is a family history of a similar skin condition, and he is unclear if psoriasis. Initial physical exam showed stable vitals, normal pulses in the lower extremity, a moderate effusion in the left knee with tenderness, and no skin erythema. There was pain with the range of movement of the left knee. He was able to bend the left knee between 10 and 60 degrees. The sensation of the left leg was intact. Skin examination showed prominent erythematous and white scaly plaques on all extremities and the body. No other joints were involved (figure 1). Laboratory testing on admission showed a peripheral white blood cell count (WBC) of 10.4 x 10^3/uL, c-reactive protein (CRP) 229 mg/L (0.00-5.00 mg/L), erythrocyte sedimentation rate 81 mm/hr (0-15 mm/hr), normal kidney function, negative HIV Ag/Ab test, and negative acute viral hepatitis panel. X-ray of the left knee showed effusion with tissue swelling but no bone or joint involvement. Aspiration of 40 ml of cloudy synovial fluid (SF) was performed in the emergency room (ER), and it showed 10,000 red blood cells/uL, 221,405 white blood cells/uL with 97% neutrophils.

**Figure 2:**
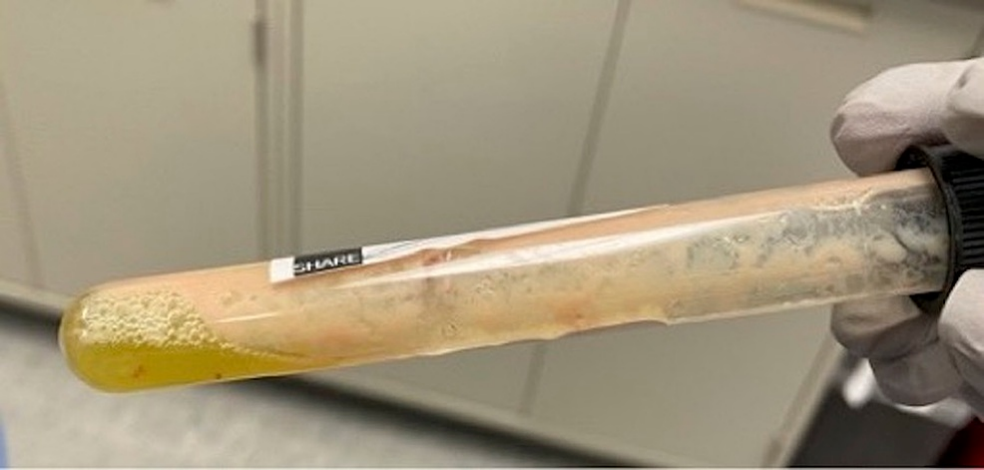
Synovial fluid: pus can be seen in the tube.

Orthopaedic surgery proceeded with irrigation and debridement of the left knee with synovial fluid and synovium tissue sampling. Synovial fluid appeared turbid and had 150,000 red blood cells /uL, 267,550 white blood cell/uL with 97% neutrophils (figure 2). Routine, anaerobic, gonococcal, acid-fast bacillus and fungal cultures of the synovial fluid remained negative. Gram stain, acid-fast bacillus, and fungal stains were also negative. Synovium biopsy showed extensive acute inflammation with negative fungal and Gram stains. The acid-fast stain was not performed on the synovium biopsy. One of two blood culture bottles grew micrococcus on admission. Repeat blood cultures were negative. Micrococcus was presumed to be a contaminant. TB QuantiFERON was not performed, given culture results. PCR was not performed in this patient as it is unavailable at our institution or the reference lab.

He was initially started on vancomycin which was transitioned to daptomycin for administration in the outpatient setting. He was empirically treated for four weeks. He returned to the ER 5 weeks later with similar symptoms in the same knee. Synovial fluid this time was bloody with 345,000 red blood cells/uL and 36,465 white blood cells/uL, with 98% neutrophils. He was discharged from the ER without antimicrobials. The patient, unfortunately, was lost to follow up after these two presentations.

## Discussion

A syndrome called pseudoseptic arthritis was coined by Call et al. in 1985 for rheumatoid arthritis patients who presented with a clinical picture similar to septic arthritis, i.e., high synovial fluid (SF) WBC count ranging 35,000 to 395,000 cells/uL with neutrophil predominance but remained negative for bacterial, mycobacterial and fungal cultures and without crystals [2]. These patients had negative blood cultures, no identifiable port of entry, or any other concomitant infection. Since then, there have been multiple other cases describing a similar pattern of pseudoseptic arthritis [3,4]. It is described primarily on rheumatoid arthritis (RA) and has been reported in other chronic diseases like Bechet's, gout, pseudogout, relapsing polychondritis, prosthetic joints, following immunosuppressive therapy, and intraarticular injections [3]. Incidence of culture-negative cases varies among studies and depends on factors like the quality of bacteriological investigations, frequency of gonococcal infections, and diagnostic criteria used to define SA. In a retrospective review of 398 cases from 1979-2005 by Eberst et al., SF cultures were negative in 19% of patients. These patients with negative cultures tended to be younger, less likely to have risk factors for SA, lower mortality rate, less likely fever or radiological signs of pre-existing joint disease, or signs of arthritis. Fourteen percent of these patients later developed some form of the rheumatic disease [5]. In 2011, Oppermann et al. identified 10 culture-negative cases, 50% had RA, and 20% had crystals in the fluid [3]. In this review, the white blood cell count was high, ranging from 18,000 cells/uL to > 400 000 cells/uL, all with negative Gram stains. The authors point out that reoccurrence is common, but no percentage is given, though two of the 5 cases had recurrence at a different site. In another case described by Page et al. in 2015, a male with RA had recurrent left hip pseudoseptic arthritis nine months apart. He was treated with unspecified intravenous antibiotics before making a PSA diagnosis. However, that patient required total joint replacement due to destruction [4].

There are a few issues with this management from an infectious disease standpoint. The management proposed for PSA by Call et al. in 1985 as "not to treat" can lead to substantial morbidity and mortality in some patients with and without inflammatory conditions [2]. In 1986, Dr Loge and Dr Mandell debated this premise, where both agreed that purulent joints should be treated as septic arthritis. Dr Mandell reveals that about 33% of patients had a full recovery, and 25% died [6]. Unfortunately, the statements made on that Call et al. paper in 1985 have now been used in recent articles, leading to discontinuation of antibiotics once septic arthritis is ruled out and instead of treating with anti-inflammatory therapies [3,4]. 

First, neither culture nor any current molecular test can reliably exclude SF infection [5]. Cultures often fail to grow fastidious organisms. For example, streptococcus, a microorganism of the skin flora, is the cause of septic arthritis in 28% of the cases and is challenging to grow [1]. This is due to the fast killing of the organism even with one dose of antibiotics and autolyzing, particularly Streptococcus pneumoniae [7]. Another example is the Mycoplasma species, where cultures are the gold standard. However, it is a fastidious organism that requires special media like yeast extract, which is not commonly done in the microbiology lab [8]. Many labs around the country dispose of routine media plates after 5-7 days due to the recovery of most organisms at 48 to 72 hours [9]. Disposal in this time frame could result in negative cultures. For example, synovial fluid cultures should be incubated for at least ten days to enhance the isolation of Cutibacterium acnes. This slow-growing bacterium is an important cause of shoulder joint infection [10]. Culture media in the last 30 years have remained similar. The current culture technique has a sensitivity of 67-87% for the diagnosis of septic arthritis [11].

Polymerase chain reaction [PCR] and bacterial genome sequencing, which is increasingly used in Infectious disease diagnostic testing, especially for culture-negative samples, has not shown promising results either in the diagnosis of etiology of native joint septic arthritis [11,12]. Literature review of the past ten years showed that the sensitivity of 16 s rDNA ranges from 20-70% from various body fluids and tissues. This wide range in sensitivity could be due to the use of different molecular testing kits and non-standardized databases [11-13]. In addition, false-positive results from the DNA of eradicated bacteria or free nucleic acids are a problem [13]. It is worth mentioning that the synovial fluid microbiome of bacteria and fungi is an evolving concept described in RA and osteoarthritis [13,14]. Given the lack of standardized molecular testing, its variable sensitivity, and the current evidence of a microbiome, it is unclear if there is a benefit of adding PCR testing to culture-negative septic arthritis at this time. Currently, major reference labs do not provide 16 s rDNA on fluids and tissues unless the organism is grown on culture [15].

Secondly, the mortality of septic arthritis per the latest estimates is 10 to 20% but can be higher when considering comorbidities. Thirdly, morbidity, including amputation, prosthetic surgery, and functional deterioration, is seen in 33% without timely treatment [1]. There is also a potential risk of exacerbating the undiagnosed bacterial infection if immunosuppressive therapies are given.

We presented a psoriasis patient with a swollen knee that resembled septic arthritis with a negative workup. To our knowledge, after a review of the literature, there are no other cases with psoriasis and pseudoseptic arthritis. The patient was placed on intravenous antibiotics for four weeks to treat septic arthritis. Evaluation of common bacteria, fungus, mycobacteria, and gonococcus was sought but not found. It is fair to say that the inflammation was due to his underlying psoriasis, but given the morbidity and mortality of septic arthritis, we decided to treat it. Since there is no universally accepted empiric regimen in such a case, he was treated with daptomycin only with a plan to add cefepime if he worsens. 

## Conclusions

As an infectious disease specialist, this is a challenging and stimulating case. There are no current guidelines for this type of case. Septic arthritis is a complicated diagnosis usually made with a combination of history, physical and laboratory findings. Many reviews give guidance on the approach to septic arthritis; still, none exist for pseudoseptic arthritis, given that imaging, synovial histology, history, physical, and laboratory do not distinguish this entity from SA. Pseudoseptic arthritis has been termed as a diagnosis of exclusion. Determining the optimal approach to these patients is challenging; as an infectious disease physician, the recommendation for this patient would be to treat given the morbidity and mortality of untreated septic joint. 
